# Investigation of the antibiofilm capacity of peptide-modified stainless steel

**DOI:** 10.1098/rsos.172165

**Published:** 2018-03-07

**Authors:** Pan Cao, Wen-Wu Li, Andrew R. Morris, Paul D. Horrocks, Cheng-Qing Yuan, Ying Yang

**Affiliations:** 1 School of Energy and Power Engineering, Wuhan University of Technology, Wuhan 430063, People's Republic of China; 2 Institute for Science and Technology in Medicine, Keele University, Stoke-on-Trent ST4 7QB, UK; 3 School of Medicine, Keele University, Newcastle-under-Lyme ST5 5BG, UK

**Keywords:** antibiofilm, synthetic peptide, dopamine, stainless steel, surface modification, topography

## Abstract

Biofilm formation on surfaces is an important research topic in ship tribology and medical implants. In this study, dopamine and two types of synthetic peptides were designed and attached to 304 stainless steel surfaces, aiming to inhibit the formation of biofilms. A combinatory surface modification procedure was applied in which dopamine was used as a coupling agent, allowing a strong binding ability with the two peptides. X-ray photoelectron spectroscopy (XPS), elemental analysis, contact angle measurement and surface roughness test were used to evaluate the efficiency of the peptide modification. An antibiofilm assay against *Staphylococcus aureus* was conducted to validate the antibiofilm capacity of the peptide-modified stainless steel samples. XPS analysis confirmed that the optimal dopamine concentration was 40 µg ml^−1^ in the coupling reaction. Element analysis showed that dopamine and the peptides had bound to the steel surfaces. The robustness assay of the modified surface demonstrated that most peptide molecules had bound on the surface of the stainless steel firmly. The contact angle of the modified surfaces was significantly changed. Modified steel samples exhibited improved antibiofilm properties in comparison to untreated and dopamine-only counterpart, with the peptide 1 modification displaying the best antibiofilm effect. The modified surfaces showed antibacterial capacity. The antibiofilm capacity of the modified surfaces was also surface topography sensitive. The steel sample surfaces polished with 600# sandpaper exhibited stronger antibiofilm capacity than those polished with other types of sandpapers after peptide modification. These findings present valuable information for future antifouling material research.

## 1. Introduction

Microorganisms can colonize a wide variety of surfaces, including ship, food packaging, aquaculture and medical devices. In many cases, this colonization leads to the subsequent formation of biofilms. Biofilms result from the accumulation of organic molecules, microorganisms and metabolites and are ubiquitous on surfaces submerged in aqueous environments [1]. The rate of surgical infections caused by biofilm can be as high as 14%, and costs more than $250 million per year in the USA [2]. The biofilms are composed of extracellular polymeric substances secreted by bacteria. These make attachment and accumulation of microorganisms easier. The formed biofilm not only allows primary colonization to become persistent, but also prevents permeation of antimicrobials. As a consequence, immune cells and proteins are unable to infiltrate and remove them. Colonization of indwelling devices causes a major problem in the medical field. Urinary catheters have long been known to suffer problematic colonization leading to biofilm obstruction [3]. Orthopaedic replacement joints are frequently colonized. A recent survey [4] showed that 75.5% of 1027 post-implant infections were caused by staphylococcal biofilms. Biofilms also lead to the increases of both hull surface roughness and the weight of ships [5]. Surface contamination can be reduced by using well-adapted materials or surface modification [6]. Numerous researchers have demonstrated that developing new methods to modify the surface properties of existing materials is an effective and economic approach [7–9].

Stainless steel is widely used in many fields, including medical device, food processing and ship manufacture industries. Bacteria and organic residues can easily adsorb onto stainless steel surfaces leading to biofouling [7]. Surface modification is, therefore, necessary to prevent this accumulation. In recent years, peptides have been used to reduce the surface energy of metals to lower biofouling. Wong *et al.* obtained a material with low surface energy by reacting a polypeptide with stainless steel [8]. Another group investigated the factors affecting the binding capacity to stainless steel. It was suggested that ion concentration changes may be a dominant factor [9]. Davis *et al*. proved that the peptide–steel reaction led to a formal or semi-formal organic–metal covalent bond formation, because stainless steel shared electrons with the disulfide bond of the peptide. The antibiofilm ability of the peptide-treated steel was also improved due to surface physiochemical properties of the material [10]. Yang *et al.* expressed the hydrophobic membrane proteins of *Bacillus subtilis* in *Escherichia coli* using recombinant genetic engineering techniques, which can be used to produce peptides for modifying surfaces [11]. Ren *et al.* [12] reacted with 304 stainless steel using genetically engineered proteins, aiming to obtain an antibacterial steel through the increase of the hydrophobicity of the treated surface. Altogether, binding appropriate peptides to metal surfaces could alter the metal's surface properties and discourage biofouling.

Dopamine coating has recently emerged as an extremely attractive approach for single or two-step surface modification of almost all kinds of materials [13]. Dopamine is a common coupling agent, which can react with a lot of solid surfaces chemically by the oxidation and self-polymerization reaction [14–16]. Dopamine-treated surfaces also revealed moderate antibacterial capacity, which is influenced by the concentration, pH and reaction duration of dopamine to the surface [17].

In this study, a combinatory surface modification procedure (two-step reaction) was investigated in which dopamine was used as the coupling agent. Two types of synthetic peptides were sequentially bound to dopamine-treated 304 stainless steel surfaces to investigate the benefits of dopamine on the peptides' binding. The impacts of the two peptide-modified metal surfaces on the adhesion of *Staphylococcus aureus* (*S. aureus*) were evaluated. The relationship between the surface topography and the antibacterial/antibiofilm properties was correlated through multiple surface characterization assays.

## 2. Material and methods

### 2.1. Materials and reagents

AISI 304 grade annealed stainless steel sheets with a thickness of 250 µm were purchased from Wuhan steel factory, which was studied for marine facilities and medical implants [18–20]. Stainless steel plates were cut into discs of Ø10 mm, and polished using 600# coast grit sandpaper before being modified by dopamine and peptides. To compare the topographic effect, six grit size sandpapers, from 180, 240, 600, 1000, 1200 to 2000#, were used for the final step of the polish. Steel discs were cleaned with absolute ethanol (15 min), immersed in acetone (1 h) and then air-dried. Dopamine hydrochloride was used without further purification (Sigma, UK). Two peptides (P1 and P2) were synthesized by Shanghai Top-peptide Bio Co. Ltd through solid-phase peptide synthesis (details of the peptides including the sequences are listed in table 1). Tris–HCl (10 mM, pH = 8.5) buffer, crystal violet (0.1%) and absolute acetic acid were purchased from Sigma. Deionized water was obtained with a Milli-Q water purification system. All the reagents in this study were analytical grade.

**Table 1.** Sequence and properties of the two synthetic peptides. The GRAVY (Grand average of hydropathicity) value for a peptide or protein is calculated as the sum of hydropathicity values of all the amino acids, divided by the number of residues in the sequence. Higher negative values indicate more hydrophilic properties, higher positive mean stronger hydrophobicity.

peptide	sequence	GRAVY	purity
P1	A**C**TSNADNKYLPKT**C**QT (disulfide 2–15)	−0.894	>95%
P2	A**C**TFFAFFFYLPFT**C**FT (disulfide 2–15)	1.588	>95%

### 2.2. Surface functionalization of 304 stainless steel by dopamine

Different concentrations of dopamine solutions were prepared by dissolving dopamine in Tris–HCl buffer (10 mM, pH = 8.5) at room temperature into final concentrations of 5, 10, 20, 40, 80 and 160 µg ml^–1^. The polished and cleaned stainless steel discs (SS) were immersed in dopamine solutions. The dopamine treatment was performed at room temperature for 24 h with rotary shaking at a rate of 60 r.p.m. At the end of the reaction, dopamine-treated discs, denoted as SS-D, were removed from the reaction mixture and ultrasonically washed for 20 min in deionized water, followed by rinsing with deionized water to remove free polymeric dopamine and air-dried. The SS-D was stored at room temperature for characterization and further reaction with peptides.

### 2.3. Covalent immobilization of peptides

The SS-D discs were placed into 24-well plates (one sample per well) and 1 ml peptide solution (20 µg ml^−1^ in Tris–HCl buffer, pH = 8.5) was added into 24-well plates for binding peptides. The reaction was allowed to proceed at room temperature with continuous stirring (60 r.p.m.) for 24 h. The reacted discs (SS-P) were removed from the reaction mixture and rinsed with a large amount of deionized water. The surfaces were stored in a dry and dark place before subjecting to property characterization and antibiofilm tests.

### 2.4. Surface characterizations

Contact angle measurements were carried out by the Biolin contact angle instrument using One Attention software with high purity water (Millipore, Milli-Q). The contact angle was measured within 15 s for a water drop (1 µl) on the surfaces and the mean of five measurements was reported. The elemental information of the sample surfaces was further analysed by X-ray photoelectron spectroscopy (XPS, ESCALAB 250Xi/ESCALAB 250Xi). The resolution of the instrument was 0.45 eV for all elements except for He, and the scan step was 0.12 eV. Each sample was scanned 12 times, and the final XPS spectra were generated by the software within the instrument.

The surface textures were analysed by the LI-3 surface profile measuring instrument (SPI, Huazhong University of Science and Technology, China). Two key surface roughness parameters, roughness average (*Sa*) and root mean square height (*Sq*) were measured and calculated to describe surface microstructures of samples and the amplitude/height distribution of the surface. *Sa* gives a good general description of height variations; *Sq* represents the standard deviation of the distribution of surface heights. The lateral and vertical resolutions for the surface profile measurement were 0.1 and 0.2 µm, respectively. The sampling area was selected as 0.8 × 0.8 mm and each step of the scan was set to 0.02 µm. The measurements were collected in three different locations for each sample. The surface parameters were derived from the collected data by the calculation programme of SPI. Ultra-deep three-dimensional micro-system (VHX-2000), which has a large depth of field, was used to record the topography of the polished discs.

### 2.5. Bacterial adhesion and bactericidal tests

The two bacterial strains, *S. aureus* (ATCC 12600) and *E. coli* (ATCC PTA-10989), the most common biofilm-producing Gram-positive and Gram-negative organisms and being extensively studied for marine environment and biomedical implants previously [21–24], were selected for the bacterial adhesion tests. Adhesion tests were performed on cells in the early stationary growth phase. *S. aureus* and *E. coli* strains were cultivated in TSB medium (Tryptic Soy Broth, Aldrich) and LB broth (Luria-Bertani, Aldrich), respectively. Bacterial strains (1 × 10^6^ CFU ml^−1^) were cultivated with three consecutive batch cultures at 37°C with shaking (180 r.p.m.). Experiments were conducted using cultures at stationary phase.

To conduct bacterial adhesion tests, samples (untreated, SS-D, SS-P) were put into 24-well suspension culture plates (one sample per well). Bacterial solutions (1 × 10^8^ CFU ml^−1^ and 1 ml well^−1^) were added to the wells. Plates were covered and sealed with Parafilm®. Samples were incubated at 37°C with continuous shaking (60 r.p.m.) for 16 h. Medium and planktonic/non-adhered bacteria were removed and plates were rinsed at room temperature three times with deionized water. In total, 300 µl of 0.1% crystal violet solution was added to each well. Plates were stood for 10 min for staining. Crystal violet solutions were discarded after staining and the discs were washed six times using sterilized water. Discs were air-dried for 1 h in a laminar flow hood. The total stain was quantified by the extraction of crystal violet by addition of 1 ml 30% acetic acid per well. Absorbance values of sample elutions were recorded at 590 nm by a plate reader (BioTek Instruments, USA). For testing the durability and robustness of the peptide-modified surfaces, SS-P1 and SS-P2 were immersed in distilled water for 2, 12, 24, 48 and 96 h with a gentle shaking (20 r.p.m.), respectively. The samples were air-dried and then were used to conduct the antibiofilm assay.

To conduct the bactericidal test, bacterial solutions were centrifuged (5000 r.p.m. for 20 min) and re-suspended in PBS solution (pH 7.4) and TSB medium, respectively. Sample surfaces were covered by 100 µl bacterial solutions (1 × 10^5^ CFU ml^−1^ in PBS and TSB). After incubation at 37°C for 3 h, samples were washed three times with autoclaved water. Samples were then immersed in 2 ml sterilized PBS solutions and sonicated for 5 min. Decimal dilutions (10^−1^ and 10^−2^) of the resulting bacterial suspension were each spread onto TSA (Tryptic Soy Agar, Aldrich) plates. After incubation for 24 h at 37°C, the number of colonies was counted. Untreated sample was used as a control. The antibacterial property was evaluated according to the following equation:

$$E=\left[\frac{N_{1}-N_{2}}{N_{1}}\right]\times 100\%,$$

where *E* is the antibacterial efficiency; *N*_1_ is the number of bacteria on untreated samples; *N*_2_ is the number of bacteria on peptide-modified surfaces.

### 2.6. Statistical analysis

All quantitative measurements were conducted on at least four samples per group. The data were represented as a mean value ± s.e.m. The group differences were analysed by one-way analysis of variance (ANOVA). *p* ≤ 0.05 was considered statistically significant. Statistical significance is indicated graphically at two levels: *p ≤ 0.05, **p ≤ 0.01.

## 3. Results

### 3.1. Peptide design and synthesis

The 16-mer peptide 1 (P1) (table 1) with an internal disulfide bond between residue 2 and 15, identical to part of the binding domain of *Pseudomonas aeruginosa* (*Pa*), can react with metals and shows antimicrobial properties [25]. P1 was a macrocyclic peptide with a number of hydrophilic and two positively charged residues enabling it to be highly hydrophilic with a negative GRAVY value (table 1). By contrast, the peptide 2 (P2) was derived from P1 by replacing seven hydrophilic amino acid residues of P1 with the hydrophobic, aromatic amino acid, phenylalanine (Phe, F), in order to improve its hydrophobicity. The GRAVY of the Phe-enriched P2 was calculated as + 1.6 indicating highly hydrophobic (table 1). Both peptides were synthesized by Fmoc-based solid-phase peptide synthesis followed disulfide formations, and purified by preparative high-performance liquid chromatography with high purities (more than 95%).

### 3.2. SS-D preparation and characterization

Dopamine was used as a coupling agent that was bound to the metal disc first, and then tethered to antibacterial peptides. Dopamine concentration affected the degree of reaction and resulted in different surface modifications. 304 stainless steel reacting with dopamine under different concentrations was studied first. It was found that, after reaction with dopamine, all treated surfaces of the discs were covered by dark or brown-coloured layers. The darkness increased considerably at higher concentration of dopamine and most thick and dark layers were easily removed during the rinsing step.

**Figure 1.**
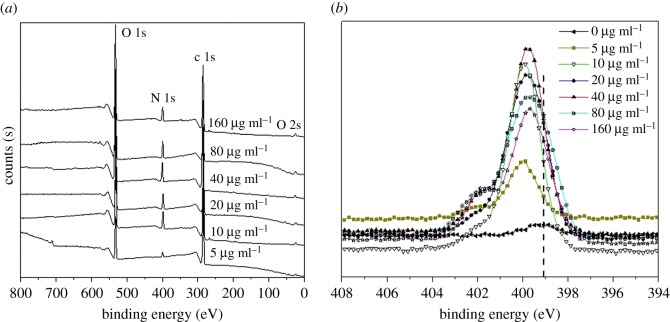
XPS spectra of SS-D samples generated by different concentrations of dopamine solutions, denoted as the values in figure (µg ml^−1^). (*a*) Survey spectra; (*b*) N1s spectra.

To confirm the presence of dopamine polymerization products on the treated discs, XPS spectra of sample surfaces were collected to quantify elemental nitrogen. The XPS survey spectra in figure 1*a* show that there were no Fe2p peaks (around 711 eV and 724 eV) in the samples when used dopamine concentrations were larger than 10 µg ml^−1^, while a small peak at around 711 eV appeared in the sample reacting with 5 µg ml^−1^ dopamine. This suggested that the dopamine-grafted layer was beyond 10 nm thickness and no iron signal underneath was detected for the samples reacting with dopamine solutions in concentrations larger than 10 µg ml^−1^. Figure 1*b* shows the characteristic peaks of N1s, and it can be seen that the peak intensities in the samples were dopamine concentration-dependent. The N1s peaks of SS-D XPS spectra shifted when compared with untreated samples. The N1s peaks at 399.8–400.2 eV were due to the presence of amine groups and the intensities of the peak increased with the increase of the used dopamine concentration then reduced when the dopamine concentration increased further. Differences in binding energies were ascribed to differences in dopamine oxidation intermediates.

**Figure 2.**
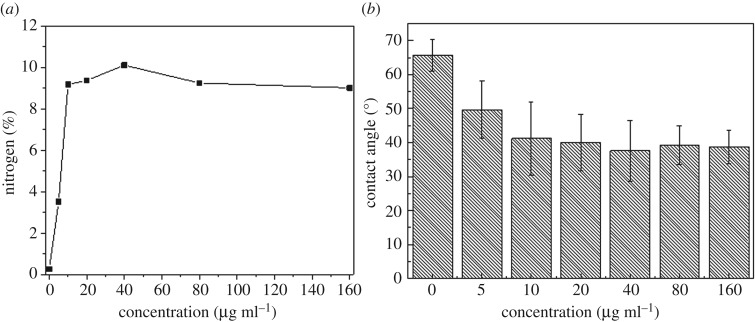
Surface characterization of SS-D samples generated by different concentrations of dopamine solution. (*a*) The content of nitrogen by XPS measurements; (*b*) contact angle.

It was apparent that the amount of dopamine bound to substrate materials depended on dopamine concentration. The bound dopamine reached maximum when its reaction concentration reached 40 µg ml^−1^. XPS examination of the content elemental nitrogen on the samples confirmed this observation (as shown in figure 2*a*). Nitrogen percentage in dopamine and dopamine oxidation intermediates was around 9.1–10%, which was consistent with the results generated by XPS analysis. The highest value of nitrogen content was detected in the samples reacting with dopamine solution at a concentration of 40 µg ml^−1^.

Bound dopamine was detected indirectly by the contact angle measurement. After reaction with dopamine, a decrease of water contact angles of the stainless steel surfaces was observed (figure 2*b*). As dopamine is a hydrophilic molecule, the contact angle of surfaces would decrease if the surface was bound by dopamine. The contact angle of untreated sample was 65.7°, while contact angle of SS-D was about 40° when using dopamine solutions with concentrations larger than 10 µg ml^−1^ (figure 2*b*). It was confirmed that the optimal reaction concentration was about 40 µg ml^−1^. Consequently, the following experiments used dopamine solutions at a concentration of 40 µg ml^−1^.

### 3.3. SS-P characterization

**Figure 3.**
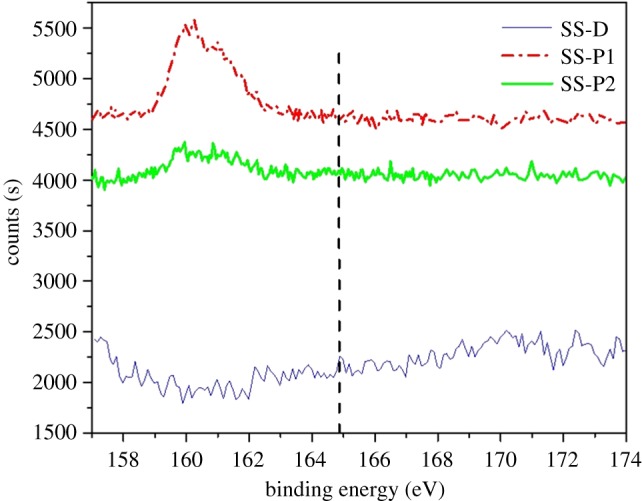
XPS spectra of SS-D, SS-P1 and SS-P2 samples.

Figure 3 shows the S2p orbital of SS-P and SS-D XPS spectra. The 304 steel contained negligible amounts of sulfur and there were no classical sulfur electron orbitals detected on SS-D by XPS. Both P1 and P2 contained two sulfur atoms within the disulfide bond that were detected by XPS. The characteristic peaks (figure 3) of S-S displayed near the location of S2p (dotted line) in the spectra strongly suggested that peptides were bound on the surfaces [10].

**Figure 4.**
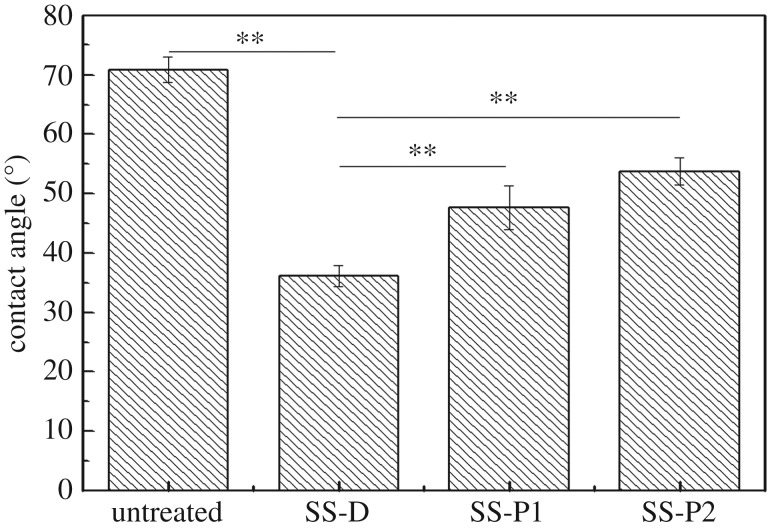
Contact angles of untreated and treated 304 stainless steel samples.

Contact angles of untreated SS-D and SS-P samples are presented in figure 4. The contact angle of the 304 stainless steel was decreased through dopamine treatment, while the contact angle increased after modifying with the peptides. The contact angle of stainless steel treated by P2 was higher than that by P1 treatment, which matched their GRAVY values (table 1) in which P2 possesses stronger hydrophobic property than P1.

### 3.4. Bacterial adhesion

#### 3.4.1. Bacterial selection

**Figure 5.**
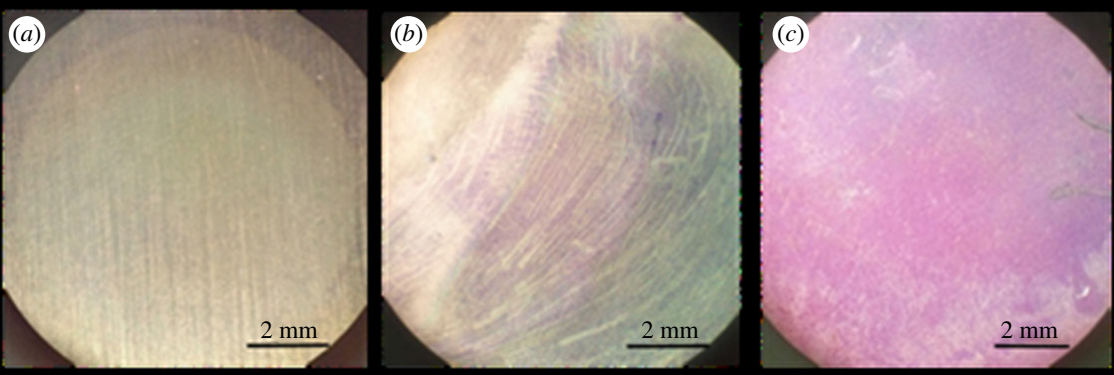
Crystal violet staining images of blank 304 stainless steel discs before and after incubating with the bacteria. (*a*) Without incubation; (*b*) incubating with *E. coli*; (*c*) incubating with *S. aureus*.

*Staphylococcus aureus* and *E. coli* are common biofilm-producing Gram-positive and Gram-negative bacteria, respectively, and are ubiquitous in nature. To assess the ability of these species to undergo bacterial adhesion and biofilm formation, untreated discs were immersed into *S. aureus* and *E. coli* solution at 37°C with gentle shaking (60 r.p.m.) for 16 h. After the incubation, the samples were stained using crystal violet. Optical images were obtained and shown in figure 5.

A layer of pure biofilm formed on the stainless steel sample surface incubation with *E. coli* (figure 5*b*) and *S. aureus* (figure 5*c*). As crystal violet intensity is proportional to the quantity of biofilm, the images revealed that the *S. aureus* strain had greater biofilm forming ability on the surface of stainless steel than the strain of *E. coli*. As *S. aureus* had a better capacity for the formation of biofilm in the study, it was used in the following bacterial adhesion assays.

#### 3.4.2. Antibiofilm activity

**Figure 6.**
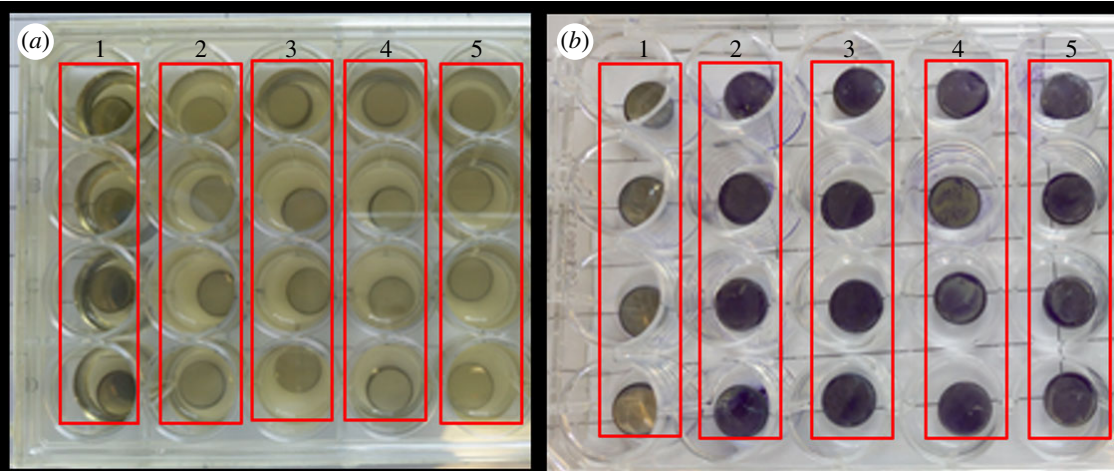
Attachment assay of *S. aureus* on different samples. (*a*) Images of bacterial solution after 16 h incubation; (*b*) images of crystal violet-stained samples. Lane 1: dopamine-treated specimens immersed into TSB; lane 2: dopamine-treated specimens immersed into bacterial solution; lane 3: untreated specimens immersed into bacterial solution; lane 4: peptide 1-treated specimens immersed into bacterial solution; lane 5: peptide 2-treated specimens immersed into bacterial solution.

**Figure 7.**
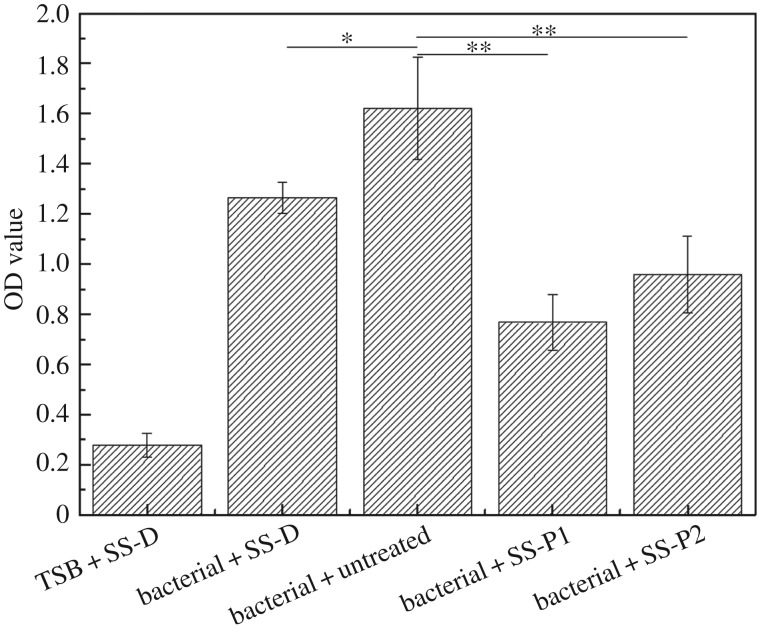
OD values of acetic acid solutions eluting from crystal violet staining assay showing the effect of the surface treatments on quantity of formed biofilm (derived from figure 6*b*).

After incubation with *S. aureus*, the transparency of remaining bacterial solution within plates changed from clear to turbid in different groups as shown in figure 6. The solutions without bacteria were still clear and the other group solution was relatively turbid, indicating that the TSB culture solution was not or less contaminated by bacteria. The solution colour of groups 2 and 3 was darker than that of groups 4 and 5, which indicated that the sample surface of groups 2–5 produced different degree of biofilms. Quantification of the biofilms using crystal violet staining (figure 7) showed that the untreated surface of sample was colonized easily by bacteria, resulting in the formation of a biofilm with a high value of staining (OD value). The lower OD value of dopamine-treated samples than that of untreated one proved that dopamine had moderate antibiofilm ability. The OD values in the treated samples with the peptides decreased significantly compared with untreated samples, indicating lower bacterial adhesion or lower biofilm formation on the treated samples. P1-treated steel surfaces showed lower OD value than P2, implying the higher antibiofilm abilities of P1-treated samples than that of P2-treated samples under the current treatment condition. Both surfaces showed stronger antibiofilm abilities than dopamine treatment alone.

**Figure 8.**
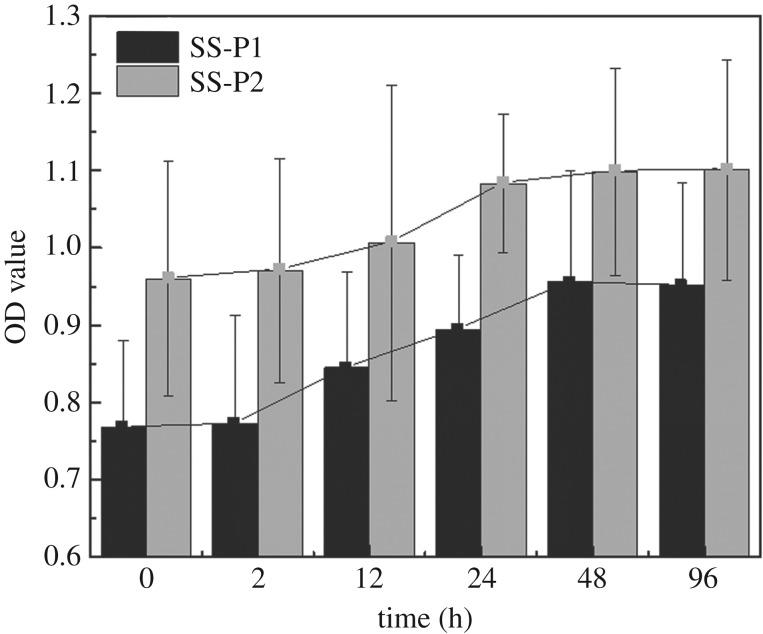
OD values of acetic acid solutions eluting from crystal violet staining assay showing the antibiofilm robustness of the peptide-modified surfaces after extra hours of immersion/shaking treatment in distilled water.

In this study, the robustness assay of the modified surface has been taken to test the stability of the resultant modified surfaces and associated antibiofilm capacity (figure 8). The OD values in elution of crystal violet increased with increasing immersion time of the samples. The increase of OD value was probably due to the loss of some of bound peptides and subsequently antibiofilm capacities after the samples were subjected to liquid immersion and shaking. Extending such treatment from 24 to 48 and 96 h did not change OD value significantly (statistical data included in the electronic supplementary material, table S1), and the treated surface showed that the OD values were still lower than that of SS-D and untreated samples. These outcomes indicated that the remained peptides on the sample surfaces were robustly bound on the surface and still functional.

#### 3.4.3. Antibacterial activity

**Figure 9.**
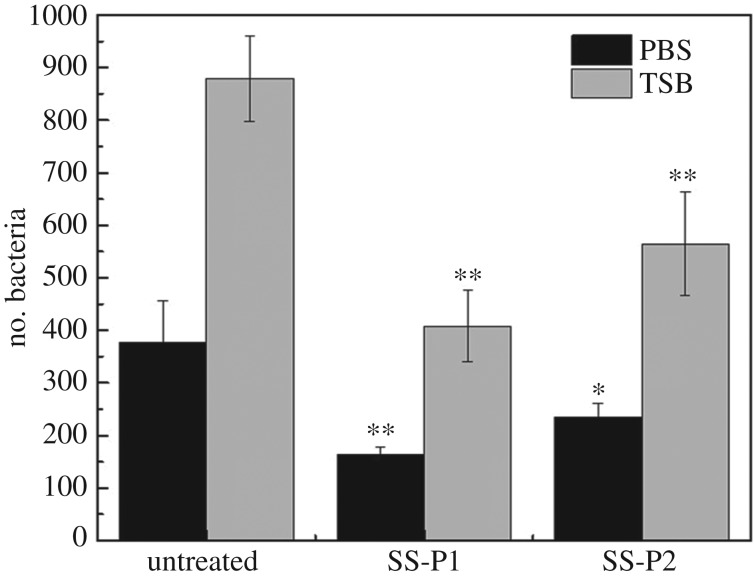
Antibacterial capacity tests for the peptide-modified surfaces, SS-P1 and SS-P2, and the effect of culture medium, PBS and TSB, on the antibacterial capacity.

The resistance capacity of the peptide-treated surface to *S. aureus* was assessed by counting bacteria colony (figure 9) in incubation. After 3 h incubation, the antibacterial efficiency of SS-P1 was approximately 56.9% and 53.6% against *S. aureus* in PBS and TSB, respectively. The antibacterial efficiency of SS-P2 was approximately 37.6% and 35.7% against *S. aureus* in PBS and TSB, respectively. The results suggested that SS-P1 and SS-P2 possessed the resistance to *S. aureus* in PBS and TSB, and the antibacterial capacity of SS-P1 was higher than SS-P2 when using the same peptide grafting reaction conditions. The number of bacteria in TSB was larger than in PBS, which was ascribed to TSB supplying nutrition for bacteria growth and proliferation, while PBS could only maintain the osmotic pressure of bacteria. (figure 9).

#### 3.4.4. Surface morphology analysis

**Figure 10.**
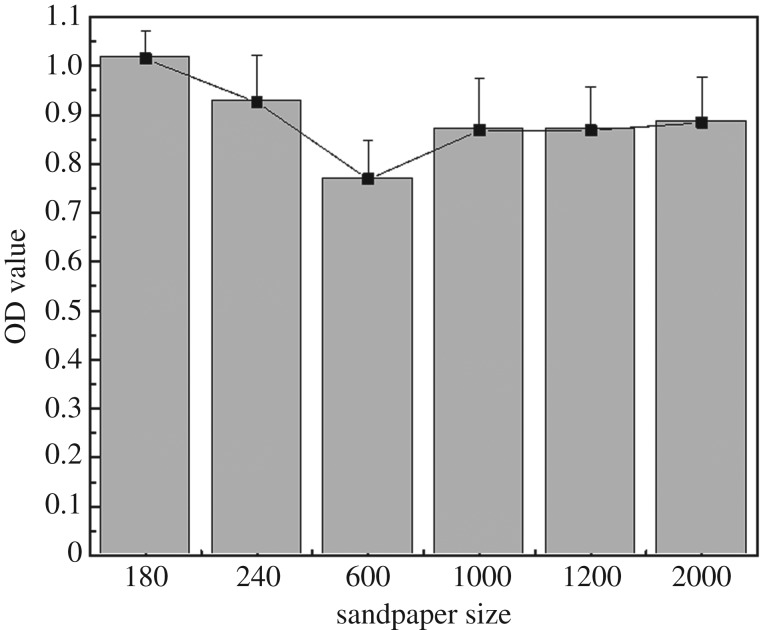
OD values of acetic acid solutions eluting from crystal violet staining assay showing the effect of sample surface roughness (polishing by different coast grit sandpaper, 180#, 240#, 600#, 1000#, 1200# and 2000#) on antibiofilm capacity after binding with peptide 1.

To study the effects of sample surface morphology due to polishing granularity on peptide binding and antimicrobial adhesion efficiency, steel discs were polished by sandpapers of 180, 240, 600, 1000, 1200 and 2000#, respectively, for the last polishing step and P1 peptide was bound to the surfaces for the antibiofilm test. Crystal violet staining after incubation showed that the 600# sandpaper-treated surface had lowest OD value. The results demonstrated that resistance capacity to *S. aureus* biofilm formation was influenced by surface morphology (figure 10), and 600# sandpaper-treated surface showed the greatest efficacy against *S. aureus* attachment.

**Figure 11.**
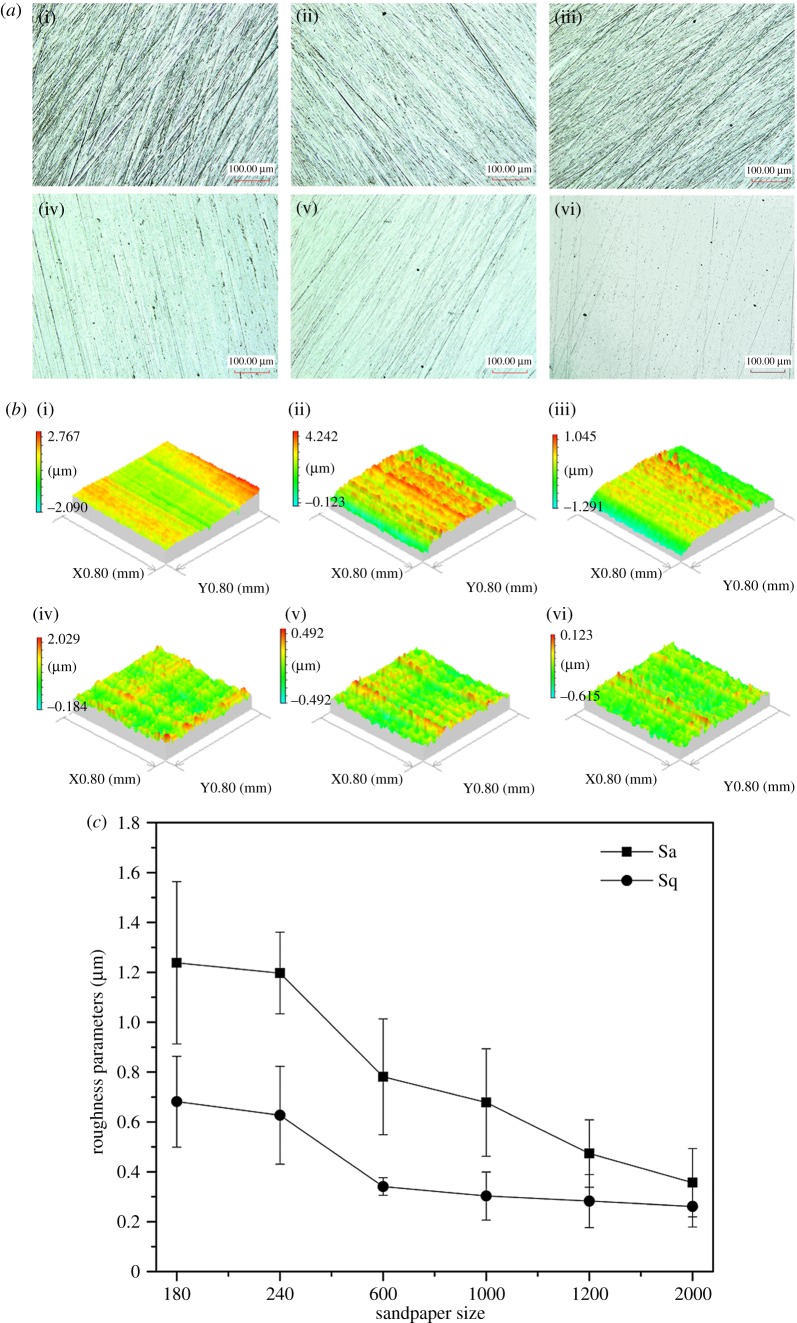
Characterization of the metal surfaces. (*a*) Surface profile images of the peptide-treated samples by ultra-depth three-dimensional micro-system, (*b*) SPI-simulated images and (*c*) Surface parameters of *Sa* and *Sq.* (i)–(vi) indicates the samples polished by the sandpaper 180#, 240#, 600#, 1000#, 1200# and 2000#, respectively.

The ultra-deep three-dimensional micro-system images and SPI-simulated image models (figure 11*a,b*) showed that the sample texture following polishing with fine sandpaper was significantly smoother than that by coarse sandpaper. The two surface profile parameters, *Sa* and *Sq*, calculated by SPI (figure 11*c*) further confirmed that discs polished with 2000# sandpaper were smoother than those polished with smaller size papers. Three-dimensional morphology models were also obtained by simulation software of SPI (figure 11*b*). The 600# sandpaper-polished surface seems to possess the proper pattern to bind more peptides and consequently reduce bacterial attachment more efficiently. The size of each individual *S. aureus* is approximate 0.8 µm, which is slightly larger than the value of *Sa* of 600# sandpaper-polished surface. This result was consistent with attachment point theory [26,27].

## 4. Discussion

Although biomaterials yielded by reaction of peptides with metals have been reported previously [28,29], low efficiency and complicated production procedures have limited their development. Dopamine coating is an attractive approach for two-step surface functionalization of almost all kinds of materials, allowing easy introduction of active groups, for example, amino- or other similar function groups [30]. In this study, a two-step coating protocol to reinforce the antibiofilm properties of stainless steel surface was explored. Dopamine was used, firstly to activate sample surface as a coupling agent and then two specifically designed antimicrobial peptides (P1 and P2, table 1) were used to modify dopamine-treated samples. SS-D, SS-P1 and SS-P2 were prepared after modification by dopamine and peptides, respectively, and both of them showed antibiofilm and antibacterial properties. Studies on the effect of the topographic features of metal surfaces on the antibiofilm adhesion illustrated that surfaces polished by 600# sandpaper were the best in the antibiofilm capacity.

During the coupling reaction with dopamine, poly-dopamine (PDA) was generated by the oxidation and self-polymerization reaction of dopamine in weak alkaline solution under an oxygen-enriched environment [31]. The accurate bio-adhesive mechanisms on the interface of substrates still remain uncertain because of the complexities of the structural analysis of PDA and its analogous molecules [32,33]. There were many intermediates (semiquinone, quinone, orthoquinone, etc.) from electron transfer among iron ions, oxygen and dopamine in the process of monomer dopamine oxidation [34]. However, PDA and dopamine contain functional groups like catechol and phenethylamine which allow them to adhere to almost all kinds of materials via non-covalent or covalent bonding [35–37]. XPS spectra, elemental analysis and contact angle measurement of dopamine-treated samples cross-validated that dopamine adhered to the surfaces of the stainless steel. In this study, the optimal reaction concentration for dopamine solutions was found to be 40 µg ml^−1^. This was because, at higher concentration, the self-polymerization reaction of dopamine would be dominated, which prevented the formation of stable binding of dopamine to metal surfaces. Both dopamine and PDA are hydrophilic molecules, and dopamine-treated surfaces became hydrophilic even if there was only PDA on the surface. That may explain the similar and low contact angle values for all dopamine-treated discs when dopamine concentrations were greater than 10 µg ml^−1^ (figure 2*b*).

XPS analysis was confirmed as a sensitive tool to study surface modification and distinguish the different surface treatments by analysis of S2p orbitals of sample surfaces. There were no classical sulfur electron orbitals in stainless steel and dopamine. The strong peaks that appeared at 160 eV region of the SS-P1 and SS-P2 spectra suggested that peptides were bound on the surface (figure 3).

The contact angle, which evaluates the wettability of the sample surfaces, can be used to assess the success of surface modification. After dopamine treatment, the contact angle of stainless steel was decreased because dopamine possesses good hydrophilicity. The contact angle increased after modification by two peptides, which indirectly indicated that peptides have bound to the metal surfaces because the peptides have lower hydrophilicity (table 1). P2-treated samples showed a higher contact angle than P1-treated samples, because P2 has more hydrophobic amino acids (e.g. Phe) and a much higher GRAVY value (table 1).

The strain of *S. aureus* used in this study showed a stronger biofilm formation ability on stainless steel surface than the strain of *E. coli* (figure 5). Thus, the bacterial adhesion experiments were performed on the modified surface with suspensions of *S. aureus*. The SS-D and SS-P surfaces showed a significant decrease of bacterial adhesion and biofilm formation after exposure to *S. aureus* compared with untreated samples (figures 6 and 7). The bound dopamine slightly reduced the amount of biofilm, indicating that dopamine has moderate antibiofilm ability, which is consistent with the previous report [17]. The overall antibacterial capacity of peptide-treated steel samples depends on the peptide's antibacterial capacity and its quantity grafted on the surface. Our results confirmed that both P1 and P2 had antibacterial capacity. The calculation of the integrated area of S2p peak in XPS spectra showed that there was around 2.5-fold higher integrated area of S2p peak of SS-P1 than that of SS-P2 (figure 9), which indicated that P1 had higher reactivity to dopamine-modified steel surface than P2 under the same reaction condition. Hence it is likely that the higher quantity of P1 peptide bound on the surface, and led to its better antibacterial capacity than P2. Such difference may be attributed to different conformations and types of the amino acid residues in the polypeptides. The higher content of cationic lysine (K), and hydrophilic serine (S) and threonine (T) in P1 than P2 were probably beneficial to the higher reactivity to the coupling agent and the relatively stronger antibiofilm effect obtained [38–40].

The robustness of SS-P was also investigated by immersing peptide-treated samples into distilled water with gentle shaking. There was no change in biofilm quantity after 48 and 96 h treatment, suggesting that most of the peptide molecules were bound on surface firmly. Only homopolymerization of dopamine, peptide or physically trapped peptides, which were weakly bound, were washed off after immersion and sonication.

The parameters of the metal surface profile were crucial to understanding biofilm formation and bacterial adhesion behaviour on them. We introduced surface morphology differences by a simple polishing protocol in this study to investigate the effect of rugosity on biofilm formation. We observed that 600# sandpaper-polished surface had the best antibiofilm capacity. The underlying mechanisms could be twofold. Firstly, the 600# sandpaper-polished surface provided more potential contact sites for dopamine and peptides during the reaction, leading more molecules to be bound to the surface. Secondly, the roughness of the 600# sandpaper-polished surface may match the resistance size of topography for *S. aureus* according to the attachment point theory of bacteria [27,41]. It has been reported that natural topographic features can resist bacterial adhesion [42,43].

Peptides rich in lysine (K), serine (S) and threonine (T) residues could bind into the PDA layer via covalent bonds [38–40]. PDA/peptide binding efficiency depends on the texture of the metal surface. Further research is required to investigate the relationship of a specific structure of peptides, surface topography of substrates and antimicrobial antibiofilm activity, which will allow better peptide design and surface microstructure of substrate to improve antibiofilm ability and binding efficiency of peptides. More aquatic or marine bacteria, such as: *Pseudomonas aeruginosa*, *Bacillus subtilis* and *Flavobacterium* spp. [44] should be tested in the future. This would better explore antibiofilm surface in antifouling application in marine environment.

## 5. Conclusion

In summary, stainless steel surface was successfully modified by two types of designed peptides *via* dopamine coupling. Dopamine formed a stable binding on the metal surface, and acted as a coupling agent to improve binding efficiency of the peptides on the surface of the stainless steel. When using the optimal concentration of dopamine solution (40 µg ml^−1^) for peptide modification of the metal surface, the samples treated with dopamine and peptides possessed antimicrobial capacity. SS-P1 was the best, followed by SS-P2, and SS-D was the last. Surface topography of the metal sample affected the adhesion capacity of bacteria on the surface of the material. The antibiofilm property of the sample surfaces polished by 600# sandpaper was best in *S. aureus* solution. Hence, the combination of peptide surface modification and topographic alteration on stainless steel materials can be a promising technique to introduce an antibiofilm feature to metal surfaces.

## Supplementary Material

The synthesis method and sequence of the peptide used in this study

## Supplementary Material

The synthesis method and sequence of the peptide used in this study

## Supplementary Material

Statistical data of robustness assay

## Data accessibility

The relevant data of this study are available in the manuscript text and the extra peptide information has been uploaded as electronic supplementary material.
