# Supplementary material for: Investigation of the antibiofilm capacity of peptide-modified stainless steel
Source: R Soc Open Sci. 2018 Mar 7;5(3):172165. doi: 10.1098/rsos.172165 (PMC5882733; doi:10.1098/rsos.172165)
Supplement: The synthesis method and sequence of the peptide used in this study [file rsos172165supp2.docx]

**Supplementary data_2**

The peptide P2 used in this study was synthesized by Shanghai Top-peptide Biological Technology Co., Ltd through solid phase peptide synthesis technique and purified using reversed-phase high-performance liquid chromatography (HPLC). Its sequence and spatial structure are shown as follows.


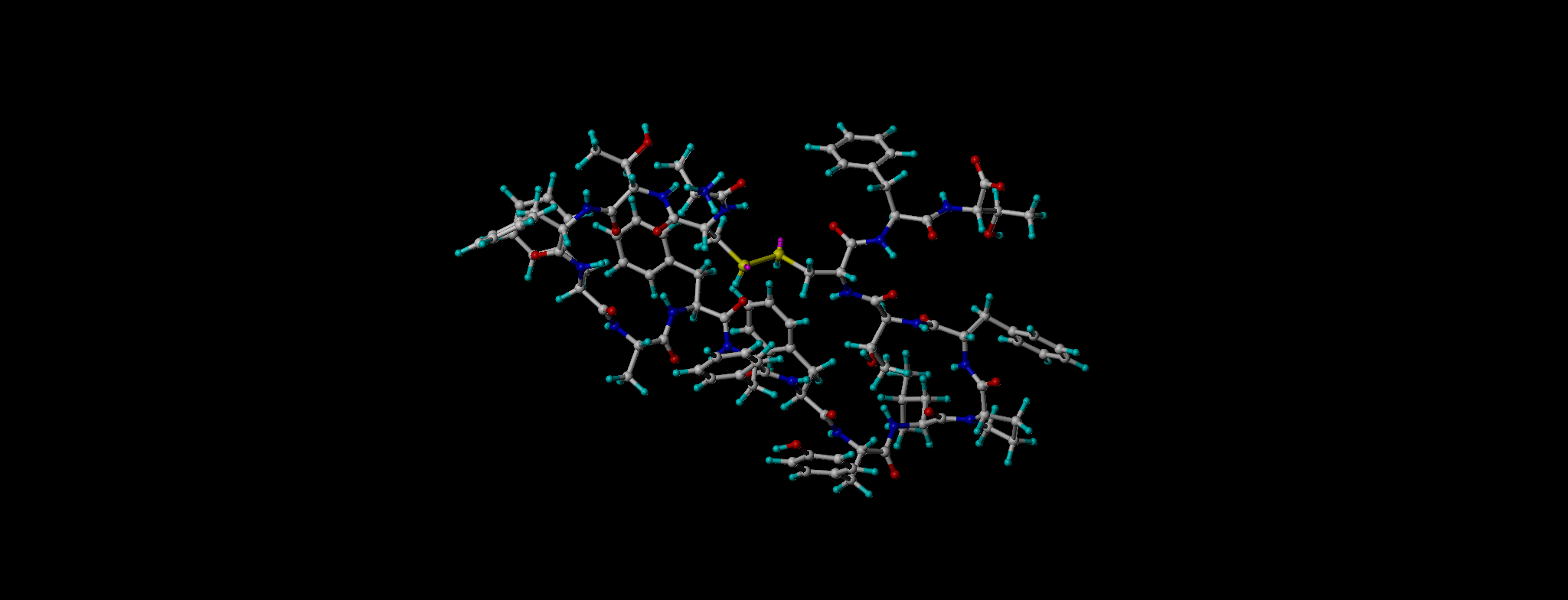


Figure 2 The sequence and spatial structure of peptide 2 (P2). The sequence of the peptide is ACTFFAFFFYLPFTCFT (disulfide 2-15) with purity of 95%.
