# Supplementary material for: Investigation of the antibiofilm capacity of peptide-modified stainless steel
Source: R Soc Open Sci. 2018 Mar 7;5(3):172165. doi: 10.1098/rsos.172165 (PMC5882733; doi:10.1098/rsos.172165)
Supplement: Statistical data of robustness assay [file rsos172165supp3.docx]

**Table 1** *P* values of the OD values for the peptide-treated samples after different treatment duration.

| **Sample** | **24 h *VS* 48 h** | **24 h *VS* 96 h** | **48 h *VS* 96 h** |
| --- | --- | --- | --- |
| SS-P1  SS-P2 | 0.573  0.585 | 0.916  0.907 | 0.965  0.989 |
